# Barocaloric effect on graphene

**DOI:** 10.1038/s41598-017-13515-9

**Published:** 2017-10-16

**Authors:** Ning Ma, Mario S. Reis

**Affiliations:** 1 Department of Physics, Taiyuan University of Technology, Taiyuan, 030024 China; 2 Department of Applied Physics, MOE Key Laboratory for Nonequilibrium Synthesis and Modulation of Condensed Matter, Xi’an Jiaotong University, Xi’an, 710049 China; 3 Institute of Physics, Fluminense Federal University, Av. Gal. Milton Tavares de Souza s/n, 24210-346 Niteroi, RJ Brazil; 4 Department of Physics and I3N, University of Aveiro, 3810 Aveiro, Portugal

## Abstract

We describe how mechanical strain is able to control the flow of heat on a graphene sheet, since this material can either absorb or expel heat from/to a thermal reservoir, depending on the strain energy. In a similar fashion as the magneto- and electro-caloric effects, the present case considers the fact that a mechanical strain produces a pseudo-magnetic field that, on its turn, is responsible for the barocaloric effect. This result pushes graphene to the list of multicaloric materials.

## Introduction

Graphene has outstanding physical properties^1^; pushing this material to the top of the list of promising quantum materials to be used in future technological devices. Just to name a few, we can cite its electrical, mechanical and thermal features, like a high electronic mobility^2^, strong mechanical resistance^3^ and, in addition, tunable thermal conductivity - from ultra-high (for heat-sinking applications), down to ultra-low values (for thermoelectric applications)^4^.

However, one special reason to justify the always fresh and renewed interest of the scientific community on graphene is the possibility of interplay of many of those outstanding, extraordinary properties. For instance, we can mention graphene plasmonics^5^, optoelectronics^6^, electro-^7^ and magneto-caloric^8,9^ effects. These last two are of great importance for the present article. Few years ago, one of us reported the interesting caloric properties of a graphene sheet due to a change of either a magnetic or an electric field; and the results were the oscillating magnetocaloric^8^ (OMCE) and electrocaloric^7^ (OECE) effects, respectively. Few years later, a follow-up of these papers appeared, discussing the properties of the OMCE of a multilayer graphene^9^ and applications into a six-stages thermodynamic cycle^10^.

In more details, those caloric effects are the ability of a material (graphene for the present case, but, of course, could be any other material^11^), to exchange heat with a thermal reservoir (under an isothermal process); or change its temperature (under an adiabatical process) due to a change of an external parameter, say, either a magnetic or an electric field. The amount of heat exchanged with a thermal reservoir, considering an isothermal process, is given by ${\rm{\Delta }}Q=T{\rm{\Delta }}S$, where ${\rm{\Delta }}S$ is the well known isothermal entropy change. The caloric effect is maximized close to the phase transition of ferromagnetic materials^11^; but, surprisingly, diamagnetic materials also present this effect (much reduced in magnitude in comparison to the former case), presenting, on the other hands, an oscillatory pattern^12,13^. These oscillations are based on the same physical mechanism as the de Haas-van Alphen^14^ and Shubnikov-de Haas effects^15^.

However, magnetic and electric external fields are not the only possible perturbations, and it is widely known that a small strain energy on graphene is enough to change its electronic properties^16^; being therefore another degree of freedom to be explored. In fact, strain produces an effective potential for the charge carriers; named as a pseudo-vector potential that, on its turn, produces a pseudo-magnetic field. These words are enough to make clear the interplay proposed in the present article: mechanical strain produces a pseudo-magnetic field that, on its turn, produces caloric properties. This is the *barocaloric effect*. In fact, multicaloric effect is a fresh issue on the literature^17^.

## Results

We have considered a graphene sheet on the *x*–*y* plane, under a strain along the three main crystallographic directions with triangular symmetry. This assumption induces a strong gauge field, that gives rise to pseudo-magnetic field ${B}_{s}$, in a similar fashion as the nanobubbles observed by Levy *et al*.^18^. The Hamiltonian for this problem is detailed described on the Methods section and has the following eigenvalues:

1 $${E}_{n,\lambda }^{\eta }=\{\begin{array}{cc}\lambda \hslash {\omega }_{s}\sqrt{n+{\mathop{{\rm{\Delta }}}\limits^{ \sim }}_{s}^{2}}, & n > 0\\ \eta \hslash {\omega }_{s}{\mathop{{\rm{\Delta }}}\limits^{ \sim }}_{s}, & n=0\end{array}$$

where *λ* = +1(−1) stands for the electrons (holes) band; ${\omega }_{s}=\sqrt{2}{v}_{F}/{l}_{s}$ is the pseudo-cyclotron frequency; ${l}_{s}=\sqrt{\hslash /|\eta e{B}_{s}|}$ is the pseudo-magnetic length; and

2 $${\tilde{{\rm{\Delta }}}}_{s}=\frac{\bar{\gamma }{B}_{s}}{\hslash {\omega }_{s}}\ll 1.$$

However, the strain energy $\bar{\gamma }{B}_{s}$ ($\approx$meV) cannot be underestimated (10 T of pseudo-magnetic field corresponds to c.a. 1 meV of strain energy)^19^. The remaining parameters above used were defined on the Methods section, when describing the Hamiltonian of the system. It is worth to note that equation 1 indicates the following: the strain does not break the sub-lattice symmetry for any non-zero Landau levels (LLs), but really removes the degeneracy of valleys on the zero-*th* LL.

From this energy spectra, one can obtain the *Grand* potential ${\rm{\Omega }}(T,{B}_{s})$, as also detailed described on the Methods section. It has two contributions: one oscillatory ${{\rm{\Omega }}}_{o}(T,{B}_{s})$ and other non-oscillatory ${{\rm{\Omega }}}_{no}(T,{B}_{s})$, in a similar fashion as found for other thermodynamic quantities for graphene^7–9^. Thus, as described on the Methods section:

3 $$\begin{array}{c}{{\rm{\Omega }}}_{no}(T,{B}_{s})=\sum _{\eta }\,[\frac{\hslash {\upsilon }_{F}}{2\sqrt{2\pi }}{(\frac{|\eta {B}_{s}|}{{\varphi }_{0}})}^{\mathrm{3/2}}\zeta (-\frac{1}{2}\mathrm{,1}+\frac{{(\bar{\gamma }{B}_{s})}^{2}}{2e\hslash {\upsilon }_{F}^{2}|\eta {B}_{s}|})+\frac{e|\eta {B}_{s}|(\bar{\gamma }{B}_{s})}{2\pi \hslash }]\\ \quad \quad \quad \quad \quad \quad \,\,\,+\frac{2{\varepsilon }_{F}{(\bar{\gamma }{B}_{s})}^{2}}{3\pi {\hslash }^{2}{\upsilon }_{F}^{2}}+\frac{2{N}_{0}}{3}{\varepsilon }_{F}\end{array}$$

and

4 $${{\rm{\Omega }}}_{o}(T,{B}_{s})=\sum _{\eta }{(\frac{|\eta {B}_{s}|}{{\varphi }_{0}})}^{2}\frac{{\hslash }^{2}{v}_{F}^{2}}{2{\varepsilon }_{F}}\sum _{k=1}^{\infty }\frac{\cos (\pi km)}{\pi {k}^{2}}\frac{{x}_{k}}{\sinh ({x}_{k})}$$

where

5 $${x}_{k}=2\pi k\frac{{{\varphi }}_{0}}{|\eta {B}_{s}|}\frac{{\varepsilon }_{F}{k}_{B}T}{{\hslash }^{2}{v}_{F}^{2}};$$

${{\varphi }}_{0}=\pi \hslash /e=2.06\times {10}^{-15}$ Tm^2^ is the magnetic flux quantum; ${N}_{0}={10}^{16}$ m^−2^ is the density of charge carriers; $\zeta (s,a)$ is the generalized Zeta function; $m={N}_{0}{{\varphi }}_{0}/|\eta \,{B}_{s}|$; and the Fermi energy reads as:

6 $${\varepsilon }_{F}=\hslash {v}_{F}{[{(\frac{\bar{\gamma }{B}_{s}}{\hslash {v}_{F}})}^{2}+\pi {N}_{0}]}^{\mathrm{1/2}}\mathrm{.}$$

It is worth to note that the strain contributes with an additional term to the Fermi energy. By contrast, the zero-strain relationship between the Fermi level and the number of charge carrier ${N}_{0}$ is simply ${\varepsilon }_{F}=\hslash {v}_{f}\sqrt{\pi {N}_{0}}$. Of course, the present effort mainly focus on a system of conserved particles, no matter whether a strain is applied or not.

From these *Grand* potentials, it is possible to obtain the entropy due to the strain; that can be evaluated using a standard thermodynamic relationship: $S(T,{B}_{s})=-\partial {\rm{\Omega }}(T,{B}_{s})/\partial T$. Considering that the non-oscillatory term (equation 3), is temperature independent (on this low temperature approximation - see the Methods section for further details), the entropy has therefore only the oscillatory contribution, that reads as:

7 $$\begin{array}{c}S(T,{B}_{s})=-\frac{\partial {{\rm{\Omega }}}_{o}(T,{B}_{s})}{\partial T}={S}_{o}(T,{B}_{s})\\ \quad \quad \quad \,\,=\sum _{\eta }\sum _{k=1}^{\infty }\frac{|\eta {B}_{s}|}{{{\varphi }}_{0}}\frac{{k}_{B}}{k}\,\cos (\pi km){\mathscr{T}}({x}_{k})\end{array}$$

where

8 $${\mathscr{T}}({x}_{k})=\frac{{x}_{k}L({x}_{k})}{\sinh ({x}_{k})}\,\,\,\,\,\,{\rm{and}}\,\,\,\,\,\,L({x}_{k})=\,\coth ({x}_{k})-\frac{1}{{x}_{k}}$$

is the Langevin function.

The caloric effect can be defined by means of the entropy change, considering the isothermal process^11^, and can be achieved measuring the difference between field- and zero-field entropies: ${\rm{\Delta }}S(T,{\rm{\Delta }}{B}_{s})=S(T,{B}_{s})-S(T,\,\mathrm{0)}$. However, it is easy to see that *S*(*T*, 0) = 0 and therefore the barocaloric effect on graphene reads as ${\rm{\Delta }}S(T,{\rm{\Delta }}{B}_{s})$ $=S(T,{B}_{s})$ (where $S(T,{B}_{s})$ is given by equation 7). Note that, at the end, only the oscillatory contribution of the entropy ${S}_{o}(T,{B}_{s})$ accounts, in a similar fashion as already discussed for the magnetocaloric effect^8^. For the sake of simplicity, as already done in other works^8^, we will only consider the $k=1$ term of the sum of equation 7. The reason of this assumption is that the hyperbolic sine function on the denominator of the equation 8 rapidly damps the summation. After these considerations, the barocaloric effect reads as:

9 $${\rm{\Delta }}S(T,{B}_{s})={k}_{B}\sum _{\eta }\frac{|\eta {B}_{s}|}{{\varphi }_{0}}\,\cos (\pi m){\mathscr{T}}({x}_{1}\mathrm{).}$$

## Discussion

**Figure 1:**
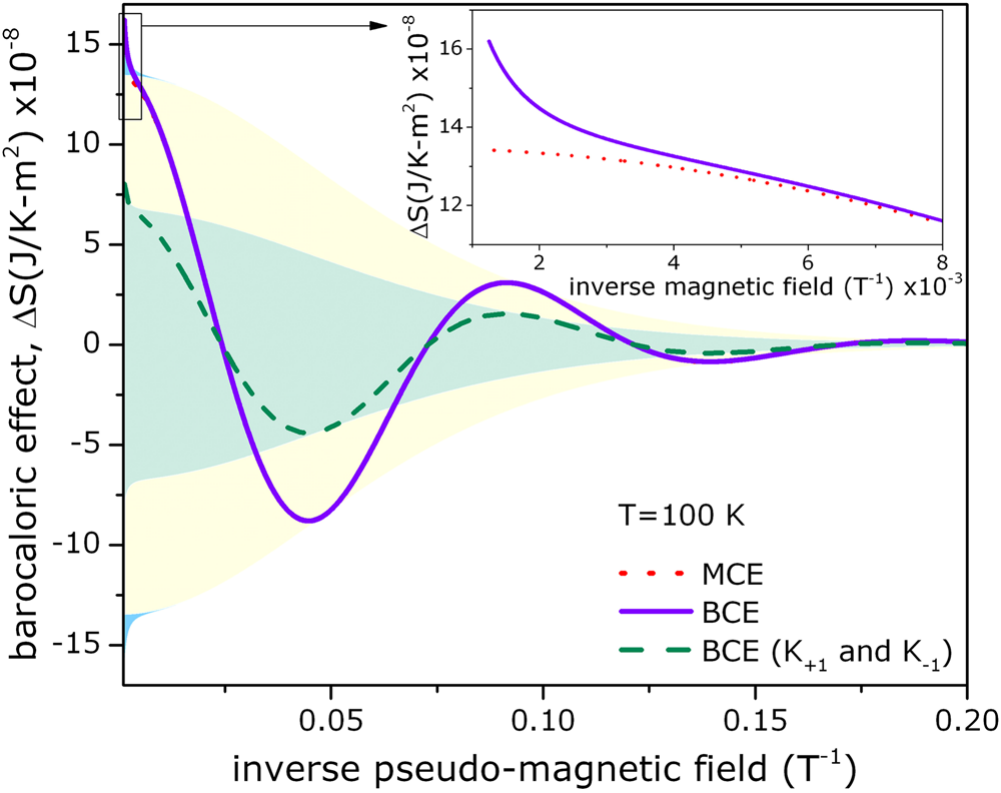
Barocaloric effect (BCE) on graphene as a function of the inverse pseudo-magnetic field - blue solid curve. Comparison with the magnetocaloric effect (MCE) on the same material - red dotted curve. The barocaloric effect considering only ${K}_{+1}$ and ${K}_{-1}$ valleys are also represented and these overlap each other (green dashed curves). Solid areas enclose the attenuation functions for those oscillations. Inset highlights the divergence, for high value of magnetic field, between MCE and BCE.

The barocaloric effect is drawn on Fig. 1 as a blue solid curve, with an attenuation given by an envelope function (yellow shadow area), that, considering $k=1$, is proportional to $|\eta {B}_{s}|{\mathscr{T}}({x}_{1})$. The first point to discuss is that the barocaloric effect does exist on graphene: if strained, it can exchange heat with a thermal reservoir; in a similar fashion as the magnetocaloric^8,9^ and the electrocaloric^7^ effects. In addition, this effect can be either normal or inverse, i.e., the material can either absorb or expel heat from/to a thermal reservoir, depending on the value of the pseudo-magnetic field, that, on its turn, depends on the strain energy. In other words, this (in/out) flow of heat can be managed by a simple handle of compression. In fact, the barocaloric effect largely overlaps the (zero-strain) oscillating magnetocaloric effect^8^ at low magnetic field, previously described by one of us, as can be seen on Fig. 1 - red dotted curve. However, there actually exists a divergence between these effects (BCE and MCE) at stronger values of magnetic field, as exhibited on the inset of Fig. 1. This anomaly must be attributed to the strain induced differences at the Fermi level, i.e., the presence of that additional ${B}_{s}$-dependent term in equation 6. However, since ${\omega }_{s}\propto {v}_{F}$, the first term (of equation 6) is therefore proportional to ${\tilde{{\rm{\Delta }}}}_{s}\ll 1$ (see equation 2). Thus, equation 6 approaches to the zero-strain case. This is the reason why the MCE and BCE matches at low magnetic field.

From another perspective, the real magnetic field hardly achieves high value as c.a. 300 T at present, but the strain pseudo-magnetic field really does^18^. One can say that the strain is able to both, either to mimic the magnetocaloric effect at low magnetic field and to produce a robust barocaloric effect. These properties can be regarded as unique advantages of strain gauge field. Based on this, more promising applications are thus eagerly expected to be realized in practice.

An important point shall be addressed on Fig. 1. We have also considered on equation 9 those different valleys ${K}_{+1}$ and ${K}_{-1}$; and, for these cases, those curves overlap each other and are represented as dashed green curves. Thus, the barocaloric effect for these cases perfectly follow the valley symmetry and therefore the strain, alone, cannot cause any valley asymmetry^19^. Although the degeneracy of the valleys in zero-$th$ energy level could be lifted by strain (as discussed above), this effect is too small to lead to a well separated valley polarisation in the barocaloric effect. Thus, the main contribution come from those all valley degenerate non-zero LLs.

**Figure 2:**
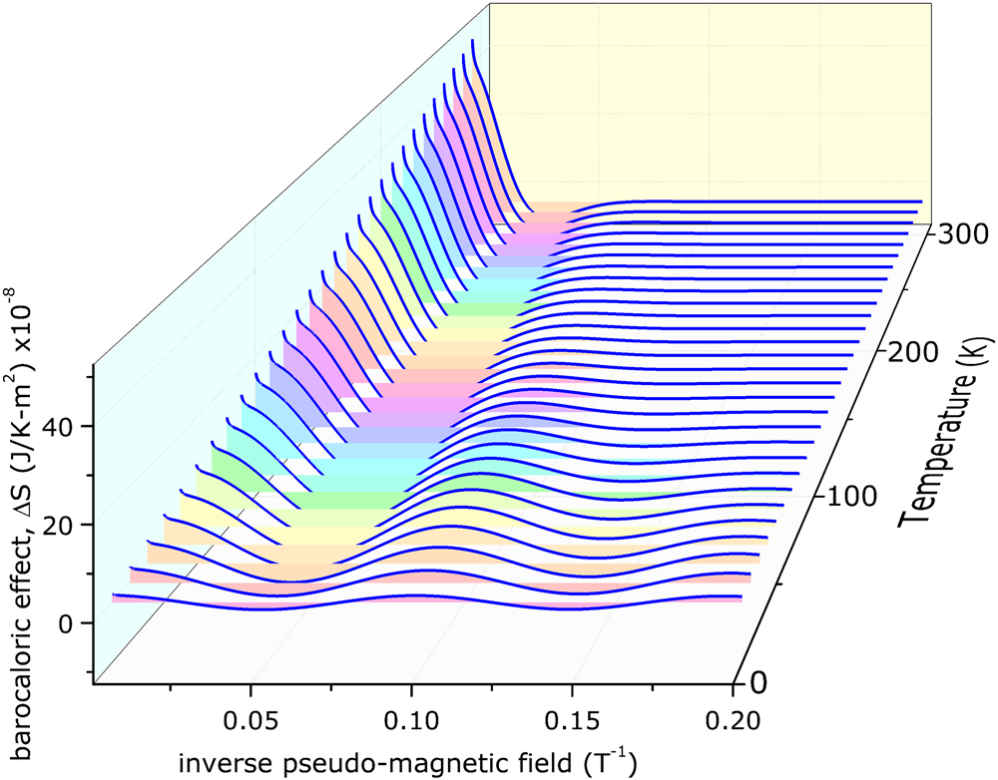
Barocaloric effect (BCE) on graphene as a function of the inverse pseudo-magnetic field and temperature. The coloured walls highlight the oscillations for each temperature, and fill the space between the curve and the zero of the graphic. The first curve represents the BCE@10 K.

After a deeper looking on equation 9, it is possible to explore the thermal dependence of the barocaloric effect. For low temperature, say 1–10 K range, the oscillations in fact exist; however, these are smashed by the temperature factor. For temperature one order of magnitude higher, say, 100 K, the oscillations are much more pronounced and the BCE much more visible. Increasing temperature up to, say, 300 K, the thermal effect damps the oscillations and these are in fact almost extinguished; however, on this temperature, the BCE reaches the highest values. These findings can be seen on Fig. 2 and, to highlight the oscillations, we have created a wall, filling the space between the BCE curves and the zero of the graphic. Final remarks on the temperature influence on the BCE: (i) for high values of pseudo-magnetic field, say, c.a. 40 T, the effect is much more prominent at higher temperatures (room temperature, for instance); and (ii) for room temperature, the BCE is already observed even for values of pseudo-magnetic field of c.a. 14 T. It is important to stress that these values are of pseudo-magnetic field (and not real magnetic field), and some experimental results already shown that is possible to achieve c.a. *B*_*s*_ =  300 T at room temperature^18^.

### Summary

We present the barocaloric effect (BCE) on graphene: the capacity of this material to exchange heat with a thermal reservoir due to a mechanical strain, like nanobubbles and even standard compression. In practice, strain produces a pseudo-magnetic field that, on its turn, changes the entropy of the material. Remarkable are the barocaloric oscillations, a pure quantum effect that allow to control the BCE as either normal or inverse, i.e., the material can either absorb or expel heat from/to a thermal reservoir, depending on the value of the pseudo-magnetic field. In other words, the flow of heat can be managed by a simple handle of compression. This effect is completely analogous to the oscillating magneto^8,9^- and electro^7^-caloric effects previously proposed for graphene, moving therefore this material to the list of multicaloric substances - a fresh issue on the literature.

## Methods

### The model

For the present work we have considered a graphene sheet on the *x*–*y* plane and subject to a strain that gives rise to a pseudo-magnetic field ${B}_{s}$ along the $z$ direction, in a similar fashion as the nanobubbles observed by Levy *et al*.^18^. The 2D Dirac Hamiltonian for this model reads as:

10 $$ {\mathcal H} =\eta {v}_{F}\vec{\sigma }\cdot {\vec{\pi }}_{\eta }-\eta \bar{\gamma }{B}_{s}{\sigma }_{z}\mathrm{.}$$

The first term of the above equation represents the kinetic energy, where: $\eta =\pm 1$ is the valley index and corresponds to two nonequivalent Dirac points (i.e. ${K}_{+1}$ and ${K}_{-1}$) in the first Brillouin zone; ${v}_{F}={10}^{6}$ m/s stands for the Fermi velocity (only 300 times smaller than the speed of light); $\vec{\sigma }$ is the pseudo-spin Pauli matrices and ${\vec{\pi }}_{\eta }=\vec{p}+e\eta {\vec{A}}_{s}$ is the canonical momentum with vector potential ${\vec{A}}_{s}$ due to the strain gauge field. Two points shall be clarified: (i) the so-called pseudo-spins have no relation with real-spins, and are introduced to denote the two carbon sub-lattices $A$ and $B$ of the graphene. These are similar to the spin index (up and down) in quantum electrodynamics and, therefore, are referred as pseudo-spins; (ii) the mentioned strain, unlike the uniaxial deformation, has been considered along the three main crystallographic directions with triangular symmetry. This assumption induces a strong gauge field, that gives rise to pseudo-magnetic field ${B}_{s}$ or, more generally, to a gauge-field vector potential ${\vec{A}}_{s}$. The second term of equation 10 is a pseudo-Zeeman coupling: the strain creates a pseudo-magnetic field that, on its turn, couples with a pseudo-spin operator. Because such a ${B}_{s}$ has opposite signs in those two valleys ${K}_{+1}$ and ${K}_{-1}$, the strain gauge field, unlike the value of the magnetic field, does not violate the time-reversal symmetry. The $\bar{\gamma }$ parameter is of the order of $9.788\times {10}^{-5}$ eV/T $\approx$ 1.7 ${\mu }_{B}$. It is interest to mention that the pseudo-magnetic field in graphene nanobubbles can achieve a magnetic field as high as 300 T, as experimentally verified by Levy *et al*.^18^: a value much higher than any other obtained in a laboratory, being therefore a unique opportunity to study a graphene sheet under an extremely high (pseudo-)magnetic field.

### *Grand* potential

The aim of this subsection is to provide further details on how to obtain the *Grand* potential, represented on equations 3 and 4. For this purpose, we start calculating the total energy of the system, at zero temperature, and then, as a second step, include the temperature effect directly on the *Grand* potential calculation.

#### Total energy

The total energy of the system at zero temperature reads as:

11 $$E=\sum _{n,\eta ,s,{k}_{y}}{E}_{n,\lambda }^{\eta }$$

and, for the present case, the above equation must be rewritten as:

12 $$E=\sum _{\eta }\sum _{n=0}^{{n}_{max}}\frac{e|\eta {B}_{s}|}{\pi \hslash }({E}_{n,\lambda }^{\eta }-{\varepsilon }_{F})+{N}_{0}{\varepsilon }_{F},$$

where we have used a trick for the first term actually corresponding to the difference between the real total energy (equation 11), and the assumed maximum energy for all of electrons occupying the Fermi level. In order to balance the physical results, we have to introduce ${N}_{0}{\varepsilon }_{F}$ as the second term. Above,

13 $${n}_{max}=[\frac{{\varepsilon }_{F}^{2}-{(\bar{\gamma }{B}_{s})}^{2}}{2e\hslash {\upsilon }_{F}^{2}|\eta {B}_{s}|}]$$

was obtained from equation 1 considering ${E}_{n,\lambda }^{\eta }$ = ${\varepsilon }_{F}$ (the square brackets [] means to take the integer). In addition, the sum over $k$ and $s$ were evaluated as:

14 $$\sum _{s,{k}_{y}}\to \frac{{L}_{y}{g}_{s}}{2\pi S}{\int }_{-{L}_{x}\mathrm{/2}{\ell }_{\eta }^{2}}^{{L}_{x}\mathrm{/2}{\ell }_{\eta }^{2}}d{k}_{y}=\frac{{g}_{s}}{2\pi {\ell }_{\eta }^{2}}$$

where $S={L}_{x}{L}_{y}$ stands for the graphene area and ${g}_{s}=2$ is the spin degeneracy.

Considering the generalised Zeta function^20^:

15 $$\zeta (z,\upsilon +k)=\zeta (z,\upsilon )-\sum _{n\mathrm{=0}}^{k-1}{(n+\upsilon )}^{-z},$$

the total energy, at zero temperature (equation 12), can be rewritten as a sum of two terms:

16 $$\begin{array}{c}{E}_{no}(T,{B}_{s})=\sum _{\eta }[\frac{\hslash {\upsilon }_{F}}{2\sqrt{2\pi }}{(\frac{|\eta {B}_{s}|}{{\varphi }_{0}})}^{\mathrm{3/2}}\zeta (-\frac{1}{2},\,1+\frac{{(\bar{\gamma }{B}_{s})}^{2}}{2e\hslash {\upsilon }_{F}^{2}|\eta {B}_{s}|})+\frac{e|\eta {B}_{s}|(\bar{\gamma }{B}_{s})}{2\pi \hslash }]\\ \quad \quad \quad \quad \quad \quad \,+\frac{2{\varepsilon }_{F}{(\bar{\gamma }{B}_{s})}^{2}}{3\pi {\hslash }^{2}{\upsilon }_{F}^{2}}+\frac{2{N}_{0}}{3}{\varepsilon }_{F},\end{array}$$

and

17 $$\begin{array}{c}{E}_{o}(T,{B}_{s})=\sum _{\eta }\{-\sqrt{2\pi }\hslash {\upsilon }_{F}{(\frac{|\eta {B}_{s}|}{{\varphi }_{0}})}^{\mathrm{3/2}}\zeta (-\frac{1}{2},\,1+\frac{{(\bar{\gamma }{B}_{s})}^{2}}{2e\hslash {\upsilon }_{F}^{2}|\eta {B}_{s}|}+[\frac{m}{2}])\\ \quad \quad \quad \quad \quad \quad -[\frac{{\varepsilon }_{F}^{3}}{3\pi {(\hslash {\upsilon }_{F})}^{2}}-\frac{{\varepsilon }_{F}|\eta {B}_{s}|}{{\varphi }_{0}}({\rm{mod}}\,[\frac{m}{2}]-\frac{1}{2})]\},\end{array}$$

where $m={N}_{0}{{\varphi }}_{0}/|\eta {B}_{s}|$; $[m\mathrm{/2}]$ stands for the integer part of $m\mathrm{/2}$; ${\rm{mod}}[m\mathrm{/2}]$ for the fractional part of $m\mathrm{/2}$. Those subscripts $no$ and $o$ for the terms above were added by convenience and this choice will be clearer later on.

The second contribution to the total energy, ${E}_{o}(T,{B}_{s})$, can be further developed. Combining the Gamma function^20^ ${\rm{\Gamma }}(n)$ with the Bernoulli polynomials^20^ ${B}_{n}(y)$, it is possible to write

18 $$\sum _{n=2}^{\infty }\frac{{\rm{\Gamma }}(n+\alpha ){B}_{n}(y)}{n!}{x}^{n}={\int }_{0}^{\infty }ds{s}^{\alpha -1}{e}^{-s}[\frac{sx{e}^{sxy}}{{e}^{sx}-1}-1-sx{B}_{1}(y)]\mathrm{.}$$

However, from ref.^20^ it is known that:

19 $$\frac{1}{{\beta }^{\upsilon }}{\rm{\Gamma }}(\upsilon )\zeta (\upsilon ,\frac{\mu }{\beta })={\int }_{0}^{\infty }\frac{{x}^{\upsilon -1}{e}^{-\mu x}dx}{1-{e}^{-\beta x}},\,\,\,\,({\rm{R}}e\mu  > \mathrm{0,}{\rm{R}}e\upsilon  > 0)\mathrm{.}$$

A comparison of these two equations leads to:

20 $$\sum _{n=2}^{\infty }\frac{{\rm{\Gamma }}(n+\alpha ){B}_{n}(y)}{n!}{x}^{n}=\frac{1}{{x}^{\alpha }}{\rm{\Gamma }}(\alpha +\mathrm{1)}\zeta (\alpha +\mathrm{1,}\,1+\frac{1}{x}-y)-{\rm{\Gamma }}(\alpha ){B}_{0}-{\rm{\Gamma }}(\alpha +\mathrm{1)}x{B}_{1}(y)$$

and then, replacing the above equation into equation 17 the term under analysis reads as:

21 $${E}_{o}(T,{B}_{s})=\sum _{\eta }{(\frac{|\eta {B}_{s}|}{{\varphi }_{0}})}^{2}\frac{\sqrt{\pi }{\hslash }^{2}{\upsilon }_{F}^{2}}{{\varepsilon }_{F}}\sum _{n\mathrm{=0}}^{\infty }\frac{{\rm{\Gamma }}(n+\mathrm{1/2}){B}_{n+2}(m\mathrm{/2})}{(n+2)!}{(\frac{2}{\upsilon })}^{n}$$

where $\upsilon ={\varepsilon }_{F}^{2}/e\hslash {\upsilon }_{F}^{2}|\eta {B}_{s}|$. Notice that the Bernoulli polynomials depend on $mod[y]$, i.e., ${B}_{n}(mod[y])$; and, for the sake of brevity, we write this as only ${B}_{n}(y)$.

The above term can be further developed. Considering now $\sqrt{e\hslash {\upsilon }_{F}^{2}|\eta {B}_{s}|}\ll {\varepsilon }_{F}$, it is possible to employ the asymptotic expansion to the Bessel function of first kind ${J}_{\alpha }(p)$; but, for this evaluation, we are only interested on $\alpha =1$ case, and therefore^20^:

22 $${J}_{1}(p)={\int }_{0}^{\infty }dt\frac{{e}^{-tp}}{\sqrt{\pi t}({t}^{2}+1)}\to \frac{1}{\sqrt{\pi }}\sum _{n=0}^{\infty }\frac{{(-1)}^{n}{\rm{\Gamma }}(n+\mathrm{1/2})}{{p}^{n+\mathrm{1/2}}}\mathrm{.}$$

On the other hand, the Bernoulli polynomials are^20^:

23 $${B}_{n}=-\frac{2n!}{{(2\pi )}^{n}}\sum _{k=1}^{\infty }\frac{1}{{k}^{n}}\,\cos (2\pi kx-\frac{n\pi }{2})\mathrm{.}$$

Inserting equations 22 and 23 into equation 21 leads therefore to the final equation for this contribution to the total energy:

24 $${E}_{o}(T,{B}_{s})=\sum _{\eta }\frac{\hslash \sqrt{\pi }}{2}{(\frac{|\eta {B}_{s}|}{{{\varphi }}_{0}})}^{\mathrm{3/2}}\sum _{k=1}^{\infty }\frac{1}{{(\pi k)}^{\mathrm{3/2}}}{J}_{1}(\pi k\upsilon )\cos (\pi km)\mathrm{.}$$

The term developed above has a cosine function and, for this reason, it is named as oscillatory contribution; while the other contribution (on equation 16), has no oscillations and therefore that is named as non-oscillatory.

#### Effect of temperature

Above we presented our results on how to obtain the total energy, at zero temperature, of the system under consideration. From now on, let us consider the temperature effect, following the methodology of refs^21,22^. The procedure is to obtain the *Grand* potential following the equation:

25 $${\rm{\Omega }}(T,{\varepsilon }_{F})={\int }_{-\infty }^{+\infty }d\varepsilon ^{\prime} {P}_{T}(\varepsilon ^{\prime} -{\varepsilon }_{F})E(\varepsilon ^{\prime} )$$

with the energy variable $\varepsilon ^{\prime}$. The distribution function ${P}_{T}$ represents, from the physical point of view, the temperature line broadening, and is obtained from the first derivative of the Fermi-Dirac distribution function $f(\varepsilon {\rm{^{\prime} }})={\{1+\exp [\beta (\varepsilon {\rm{^{\prime} }}-{\varepsilon }_{F})]\}}^{-1}$ with respect to $\varepsilon {\rm{^{\prime} }}$, as detailed on refs^21,22^:

26 $${P}_{T}=-\frac{\partial f(\varepsilon ^{\prime} )}{\partial \varepsilon ^{\prime} }=\frac{1}{4{k}_{B}T{\cosh }^{2}[(\varepsilon ^{\prime} -{\varepsilon }_{F}\mathrm{)/2}{k}_{B}T]}\mathrm{.}$$

Let us first consider the non-oscillatory contribution, and, for this purpose, we need to insert equation 16 into equation 25. Neglecting terms on ${\mathscr{O}}({k}_{B}T)$, changing the variable of integration as $\varepsilon ^{\prime} \to 2{k}_{B}T\varepsilon$ and considering the special integral properties of odd functions on an infinite interval with an energy shift as $\varepsilon ^{\prime} \to \varepsilon ^{\prime} +{\varepsilon }_{F}$, we find the non-oscillatory term of the thermodynamic potential:

27 $$\begin{array}{c}{{\rm{\Omega }}}_{no}(T,{B}_{s})=\sum _{\eta }\,[\frac{\hslash {\upsilon }_{F}}{2\sqrt{2\pi }}{(\frac{|\eta {B}_{s}|}{{\varphi }_{0}})}^{\mathrm{3/2}}\zeta (-\frac{1}{2},\,1+\frac{{(\bar{\gamma }{B}_{s})}^{2}}{2e\hslash {\upsilon }_{F}^{2}|\eta {B}_{s}|})+\frac{e|\eta {B}_{s}|(\bar{\gamma }{B}_{s})}{2\pi \hslash }]\\ \quad \quad \quad \quad \quad \quad \,\,+\frac{2{\varepsilon }_{F}{(\bar{\gamma }{B}_{s})}^{2}}{3\pi {\hslash }^{2}{\upsilon }_{F}^{2}}+\frac{2{N}_{0}}{3}{\varepsilon }_{F}\mathrm{.}\end{array}$$

Interestingly, the approximate result above for finite temperature is the same as equation 16 of zero temperature. This reveals that the non-oscillatory part of the thermodynamic potential could be considered independent of the temperature, thereby making null contribution to the present entropy change and BCE effect.

Analogously to above, let us now consider the oscillatory contribution, by considering the integral representation for ${J}_{1}(p,r)$ as below^20^:

28 $$\sqrt{\pi }{J}_{1}(p,r)=-{\rm{Im}}{\int }_{0}^{\infty }\frac{dt{e}^{-pt-r/t}}{\sqrt{t}(t+i)}$$

and then rewrite equation 24 based on the equation above:

29 $${E}_{o}(T,{B}_{s})=-\sum _{\eta }\frac{\hslash {\upsilon }_{F}}{2}{(\frac{|\eta {B}_{s}|}{{\varphi }_{0}})}^{\mathrm{3/2}}\sum _{k\mathrm{=1}}^{\infty }\frac{1}{{(\pi k)}^{\mathrm{3/2}}}{\rm{Im}}\{{e}^{-i\pi \mathrm{/4}}{\int }_{0}^{\infty }\frac{dt\exp [-i\pi k(\upsilon t+m)]}{\sqrt{t}(t+i)}\}\mathrm{.}$$

Thus, the *Grand* potential can be obtained by inserting the above equation into equation 25; and it reads as:

30 $$\begin{array}{rcl}{{\rm{\Omega }}}_{o}(T,{B}_{s}) & = & -\sum _{\eta }\frac{\hslash {\upsilon }_{F}}{2}{(\frac{|\eta {B}_{s}|}{{\phi }_{0}})}^{\mathrm{3/2}}\\  &  & \times \sum _{k=1}^{\infty }\frac{1}{{(\pi k)}^{\mathrm{3/2}}}{\rm{Im}}\{{e}^{-i\pi \mathrm{/4}}{\int }_{0}^{\infty }\frac{dt}{\sqrt{t}(t+1)}{\int }_{-\infty }^{\infty }\frac{d\varepsilon ^{\prime} }{4{k}_{B}T{\cosh }^{2}\frac{(\varepsilon ^{\prime} -{\varepsilon }_{F})}{2{k}_{B}T}}\\  &  & \times \exp [-i\pi k\frac{{\varepsilon }^{\text{'}2}(t+\mathrm{1)}-{(\bar{\gamma }{B}_{s})}^{2}}{e\hslash {\upsilon }_{F}^{2}|\eta {B}_{s}|}]\}\mathrm{.}\end{array}$$

Neglecting terms on ${\mathscr{O}}(({k}_{B}T{)}^{2})$ and, once again, considering the change of variable as $\varepsilon ^{\prime} \to 2{k}_{B}T\varepsilon$ and an energy shift as $\varepsilon ^{\prime} \to \varepsilon ^{\prime} +{\varepsilon }_{F}$, the inner integral above becomes:

31 $$\begin{array}{c}{\int }_{-\infty }^{\infty }\frac{d\varepsilon }{{\cosh }^{2}\varepsilon }\exp (\frac{-i\pi k(t+1)4{k}_{B}T\varepsilon {\varepsilon }_{F}}{e\hslash {\upsilon }_{F}^{2}|\eta {B}_{s}|})=2{\int }_{0}^{\infty }\frac{d\varepsilon }{{\cosh }^{2}\varepsilon }\,\cos (\frac{4\pi k(t+1){k}_{B}T{\varepsilon }_{F}}{e\hslash {\upsilon }_{F}^{2}|\eta {B}_{s}|}\varepsilon )\\ \quad \quad \quad \quad \quad \quad \quad \quad \quad \quad \quad \quad \quad \quad \quad \quad =\,\frac{4{\pi }^{2}k(t+1){k}_{B}T{\varepsilon }_{F}}{e\hslash {\upsilon }_{F}^{2}|\eta {B}_{s}|\sinh \,{\rho }_{\eta }},\end{array}$$

where

32 $${\rho }_{\eta }=\frac{2{\pi }^{2}k(t+1){k}_{B}T{\varepsilon }_{F}}{e\hslash {\upsilon }_{F}^{2}|\eta {B}_{s}|}\mathrm{.}$$

Above, to solve the integral, we have used the formula^20^:

33 $${\int }_{0}^{\infty }\frac{\cos \,az}{{\cosh }^{2}\beta z}dz=\frac{a\pi }{2{\beta }^{2}\,\sinh \,\frac{a\pi }{2\beta }}\mathrm{.}\,\,\,\,({\rm{R}}e\beta  > \mathrm{0,}a > \mathrm{0)}$$

The above result (equation 31) into equation 30 gives:

34 $${{\rm{\Omega }}}_{o}(T,{B}_{s})=-\sum _{\eta }\frac{\pi {(e|\eta {B}_{s}|)}^{\mathrm{1/2}}{k}_{B}T{\varepsilon }_{F}}{{\upsilon }_{F}{(e{\varphi }_{0})}^{\mathrm{3/2}}}\sum _{k\mathrm{=1}}^{\infty }\frac{1}{{(\pi k)}^{\mathrm{1/2}}}{\rm{Im}}\{{e}^{-i\pi \mathrm{/4}}{e}^{-i\pi km}{\int }_{0}^{\infty }\frac{dt}{\sqrt{t}}{e}^{-i\pi kt\upsilon }\frac{1}{\sinh \,{\rho }_{\eta }}\}\mathrm{.}$$

Now we have only one integral to solve, over $t$. Note the non-oscillating factor of the integrand has a maximum at $t=0$, and therefore we can evaluate the $sinh$ at $t=0$; while the remaining integral over $t$ is evaluated by means of the equation:

35 $${\int }_{0}^{\infty }{x}^{\nu -1}{e}^{-\mu x}dx=\frac{1}{{\mu }^{\nu }}{\rm{\Gamma }}(\nu ),\,\,\,\,({\rm{Re}}\mu  > \mathrm{0,}\,{\rm{Re}}\nu  > \mathrm{0)}$$

Therefore, we find:

36 $${{\rm{\Omega }}}_{o}(T,{B}_{s})=-\sum _{\eta }\frac{\pi {(e|\eta {B}_{s}|)}^{\mathrm{1/2}}{k}_{B}T{\varepsilon }_{F}}{{\upsilon }_{F}{(e{\varphi }_{0})}^{\mathrm{3/2}}}\sum _{k\mathrm{=1}}^{\infty }\frac{1}{{(\pi k)}^{\mathrm{1/2}}}{\rm{Im}}\{{e}^{-i\pi \mathrm{/4}}{e}^{-i\pi km}\frac{{\rm{\Gamma }}(\mathrm{1/2})}{{(i\pi k\upsilon )}^{\mathrm{1/2}}}\frac{1}{\sinh \,{x}_{k}}\}$$

with $${\rm{\Gamma }}\mathrm{(1/2)}=\sqrt{\pi }$$ and $${x}_{k}=2\pi k{{\varphi }}_{0}{\varepsilon }_{F}{k}_{B}T/|\eta {B}_{s}|{\hslash }^{2}{\upsilon }_{F}^{2}$$.

Finally, we obtain the oscillatory term of the *Grand* potential:

37 $${{\rm{\Omega }}}_{o}(T,{B}_{s})=\sum _{\eta }{(\frac{|\eta {B}_{s}|}{{\varphi }_{0}})}^{2}\frac{{\hslash }^{2}{\upsilon }_{F}^{2}}{2{\varepsilon }_{F}}\sum _{k\mathrm{=1}}^{\infty }\frac{\cos (\pi km)}{\pi {k}^{2}}\frac{{x}_{k}}{\sinh ({x}_{k})}\mathrm{.}$$
